# Evaluation of the Happiness Through Goal-Setting Training

**DOI:** 10.1177/00332941211071007

**Published:** 2022-01-27

**Authors:** Christian Ehrlich

**Affiliations:** 6395 Oxford Brookes University, Oxford, UK

**Keywords:** evaluation, goal-striving reasons framework, happiness training, subjective well-being, work engagement

## Abstract

This paper describes the evaluation of the Happiness through Goal-Setting Training, a multiple intervention approach which helps participants to reflect on, and modify their reasons for goal pursuit. The training is theoretically grounded in the goal-striving reasons framework. This framework captures four important reasons for goal pursuit and has received a substantial amount of empirical support for its predictive power in relation to positive psychological functioning. The four goal-striving reasons are the pursuit of goals out of pleasure, altruism, fear of self-esteem loss or necessity. The evaluation of the training, employing a before-and-after study design, is based on two data sets comprising data from a face-to-face delivery of the training (*N =* 41) and an online delivery of the training (*N =* 40). Overall, the findings from both studies, using paired sample t-tests, show that the Happiness through Goal-Setting Training significantly improves the quality of people’s reasons for goal pursuit as well as their positive psychological functioning measured through life satisfaction, positive affect, negative affect and work-engagement. Thus, the Happiness through Goal-Setting Training complements the existing suite of well-being interventions by providing a happiness training that focusses specifically on improving people’s reasons for goal-pursuit.

## Introduction

Research has conclusively shown that the reasons why people pursue their most important goals in life predict their subjective well-being and positive psychological functioning (Deci & Ryan, 2000; Sheldon et al., 2004). Most researchers currently employ the self-concordance theory to measure the quality of people’s reasons for goal pursuit (Sheldon & Elliot, 1999). Self-concordance theory distinguishes between autonomous and controlled reasons for goal pursuit whereby autonomous goal motivation emanates from self-choices as opposed to controlled goal pursuits which emanate from external pressures outside the person. Notwithstanding the relevance and influence of self-concordance theory for the happiness literature, it seems reasonable to question whether the autonomy-control dimension is the only relevant dimension along which to classify people’s reasons for goal pursuit. Not the least because the identification of further goal-reason dimensions allows us to identify and develop further theory-based happiness interventions that go beyond the provision of an autonomy supportive environment as derived from self-concordance theory (cf. Stone et al., 2009).

In this context, an alternative and theoretically different concept to measure the reasons for goal pursuit has been presented in the literature: the *goal-striving reasons framework (GSRF;*Ehrlich, 2012*).* The GSRF has not only been shown to be a significant predictor of people’s subjective well-being for a variety of populations ranging from undergraduate students to voluntary sector employees. It has also been shown to have, at times, higher predictive power for positive psychological functioning than the self-concordance theory (Ehrlich, 2018, 2019; Ehrlich & Bipp, 2016).

Unlike the self-concordance theory, GSRF operationalises the quality of people’s reasons for goal pursuit based on two of the most influential dimensions within motivation psychology: the approach/avoidance dimension and the internal/external dimension. Approach reasons are hereby defined as reasons for goal-pursuit that are moving towards a desired outcome whereas avoidance reasons are defined as moving away from an undesired outcome (cf. Elliot et al., 1997). Based on the ample amount of empirical support showing that people with approach goals tend to report higher levels of happiness than people with avoidance goals (Coats et al., 1996; Dickson, 2006; Ryan & Deci, 2001), the GSRF takes the notion of approach/avoidance motivation and employs it to distinguish between approach/avoidance goal reasons. Empirical research on the GSRF has conclusively shown that the application of the approach/avoidance dimension onto the goal-reason level is an important differentiation with approach goal reasons being positively correlated to happiness whereas avoidance reasons being negatively associated with happiness (Dickson, 2006; Ehrlich, 2012, 2018, 2019; Ehrlich & Bipp, 2016).

Of equal importance for our understanding of the quality of people’s goal-reasons is the internal/external dimension, which in the GSRF is further specified by employing Ford and Nichols’ (1987) distinction between within-person reasons (internal) and person-environment reasons (external). Within-person reasons are characterised as goal reasons that are predominantly aimed at consequences for the person itself in contrast to person-environment reasons which are predominantly aimed at changing the external situation. This distinction is relevant to understand the quality of people’s goal reasons as it has been shown that internal goal-reasons tend to be more strongly associated with people’s happiness than external goal-reasons (Ehrlich, 2012, 2019; Ehrlich & Bipp, 2016).

By combining the approach/avoidance dimension with the internal/external dimension, the GSRF identifies four specific goal-reasons each of which represents one of the possible four combinations. The four reasons are the degree to which people pursue their most important goals out of pleasure (approach/internal), the degree to which people pursue their goals for altruistic reasons (approach/external), the degree to which people pursue their goals to avoid any loss of self-esteem (avoidance, internal) and the degree to which people pursue their goals out of necessity (avoidance, external).

Based on the accumulating empirical support showing that people’s goal-striving reasons are an important predictor for people’s happiness alongside growing evidence for the effectiveness of positive psychology interventions for people’s well-being in general (Bergsma et al., 2020; Krekel et al., 2020), the next logical step was to develop a training approach based on the GSRF: the *Happiness through Goal-Setting Training (HTGST)*.

The development of the HTGST was hereby guided by the following considerations. To be coherent with the underlying assumption of the GSRF, the ultimate aim of the training was to identify relevant theories and interventions that can be employed to either increase the degree to which people pursue their most important goals out of pleasure and altruism or to decrease the extent to which people pursue their goals out of fear of self-esteem loss or necessity. Furthermore, it seemed, in the first instance, most beneficial to develop a training approach which focusses on the improvement of people’s work-related goal-striving reasons. This is because goals and goal-setting practices are a well-established motivational technique in the workplace (Latham, 2012) resulting in the fact that most employees find it easy to state three to four work-related goals which they currently strive for. Work-related goals have also been shown to be strongly associated with people’s psychological functioning at work (Maier & Brunstein, 2001).

In relation to the development of the HTGST, the focus of the training is on the improvement of a total of four distinct goal-reasons which means the HTGST can be classified as a multiple intervention or multi-component programme (Hendriks, Schotanus-Dijkstra, Hassankhan & Bohlmeijer, 2019). By doing so, it goes beyond the most common form of *Positive Psychology Interventions (PPIs*) which focus on one single or very few interventions in isolation (Gander et al., 2016; Lambert et al., 2019). This is important insofar as multiple interventions have been found to outperform single interventions (Bergsma et al., 2020; Hendriks et al., 2019) due to the fact that they offer more variety within their interventions preventing the occurrence of hedonic adaptation (Bergsma et al., 2020; Sheldon & Lyubomirsky 2012). Equally important in this context is the fact that, according to Lambert et al. (2019), most PPIs are typically not framed upon a theoretical orientation which prevents the integration of multiple interventions within a given training approach. To the authors’ knowledge, most of the multiple interventions trainings are based on Seligman’s (2011) PERMA model (Gander, et al., 2016; Lambert, et al., 2019). Notwithstanding the relevance of the PERMA model within the Positive Psychology literature, the focus of the PERMA model is on the application of positive emotions, engagement, etc. into one’s daily life generally and therefore differs from HTGST which provides interventions that focus exclusively on the modification on people’s reasons for goal pursuit. Consequently, the HTGST might be particularly relevant for people who live a very goal-oriented life such as employees.

Based on these observations, it can be concluded that a happiness training which integrates a multitude of relevant concepts and PPIs based on their relevance for specific goal-striving reasons as identified within the GSRF promises to offer an important addition to the happiness training literature. The aim of the paper at hand is therefore to describe the HTGST as well as presenting two evaluation studies about the effects of the HTGST for positive psychological functioning.

## The Happiness Through Goal-Setting Training

The delivery of the training content within HTGST is similar for each of the four goal-striving reasons and follows the typical structure of many PPIs (Bergsma et al., 2020). Participants are firstly introduced to the core research findings of each goal-striving reason in relation to happiness. This is followed by a discussion about how to apply this research to the participants’ own goals supplemented by practical exercises. The order in which participants are introduced to the four goal-striving reasons in the training follows the same order as that presented in the paper at hand. Prior to the discussions and exercises on any specific goal-string reasons, participants are informed about the importance of goal-reasons as a major contributor to positive psychological functioning, drawing on relevant research in this field (Carver & Baird, 1998; Deci & Ryan, 2000; Ehrlich, 2012; Ehrlich & Bipp, 2016; Sheldon et al., 2004). For a comprehensive description of the HTGST including detailed instructions for all exercises presented, please refer to Ehrlich and Milston (2022).

### Self-Esteem

The first topic discussed within the HTGST is self-esteem.^1^ Particular focus is given to the distinction between conditional and unconditional self-esteem (cf. Crocker & Park, 2004; Crocker & Knight, 2005; Ellis, 2005; Kernis, 2003; Patterson & Joseph, 2006; Rogers, 1957). Conditional self-esteem is characterised by the belief that one’s self-esteem is contingent on one’s successes or failures in life. Unconditional self-esteem on the other hand is based on the assumption that one can acquire a positive view about oneself which is not directly linked to one’s successes or failures in life (cf. Patterson & Joseph, 2006). Thus, people are in a position to maintain a positive view about themselves (high level of self-esteem) despite their flaws and imperfections through self-kindness (Neff, 2009). With regard to happiness, research has provided conclusive evidence that unconditional self-esteem is typically related to higher levels of positive psychological functioning than conditional self-esteem (Crocker & Knight, 2005; Crocker & Park, 2004; Neff, 2009).

After participants are introduced to the concept of unconditional self-esteem they are asked to reflect on the degree to which their most important goals are underpinned by their desire to validate their self-worth. The aim of this exercise is for participants to identify which of their goals are underpinned by the desire to prove one’s self-worth – typically labelled as self-validating goals (Crocker & Knight, 2005). After having identified any self-validating aspects within their goals, participants are asked to reflect on whether they could stop or decrease the urge to pursue those goals for self-validating purposes, and instead adopt a more unconditional view when pursuing those goals (e.g. ‘I can still like myself even if I would not attain goal X’). Participants are hereby given time for individual self-reflection to identify any self-validating motivation within their four most important goals. Furthermore, where deemed appropriate, they are encouraged (through cognitive reframing of how one can achieve high positive self-regard) to start to reduce the degree to which the goal is pursued for self-validating purposes.

Next to the concept of unconditional self-esteem, the HTGST also introduces participants to the concept of learning goals as another way to protect one’s self-esteem when experiencing setbacks during goal pursuit. Learning goals are hereby defined as goals where an individual is concerned with improving their skills or competencies (Dweck & Leggett, 1988) as opposed to performance goals whereby the focus is on gaining a favourable judgement of one’s capabilities through goal-attainment. The setting of learning goals is typically associated with the basic assumption that one’s capabilities are malleable as opposed to fixed in the case of performance goals (Latham, 2012). Based on this differentiation, learning goals provide a learning opportunity in the case of setbacks or failure in relation to goal pursuit (e.g. ‘I have not learned enough, yet’) rather than a self-esteem diminishing experience in the case of performance goals where failure is a direct reflection of one’s capabilities (e.g. ‘I am not good enough’). After the participants are introduced to the advantages of learning goals they are asked to reflect on their most important goals and, where applicable, translate any of their performance goals into a learning goal. This exercise on setting learning goals further contributes to participants being able to pursue their goals without the fear of self-esteem loss in case of setbacks or even non-attainment of their goals.

### Pleasure

The next goal-striving reasons within HTGST is pleasure. This goal-striving reason is very much based on the notion that the amount of pleasure or positive emotions we experience whilst pursuing our goals is strongly associated with increased subjective well-being (Brunstein, 1993; Carver & Baird, 1998; Csikzsentmihalyi, 1988; Deci & Ryan, 2000). At the same time, empirical evidence suggests that individuals quite often find it difficult to pursue goals they enjoy (Kehr, 2004; Sheldon, 2014). Within HTGST participants are introduced to some of the key research findings on the importance of positive emotions in relation to goal pursuit and happiness. Particular focus is given in relation to Fredrickson’s (2001) broaden-and-build theory of positive emotions as this lays the foundation for the first exercise on pleasure in the training. According to the broaden-and-build theory, the experience of positive emotions creates an upward spiral, making people feel more creative, more innovative, more resourceful and more optimistic (Cohn & Fredrickson, 2010; Kok et al., 2008). In the context of the HTGST, this is important as positive emotions do not simply cease when finishing a pleasurable activity but continue to have positive spill-over effects into the future. Thus, experiencing positive emotions causes people to approach or to reflect on subsequent tasks (or indeed their most important goals) in a more optimistic and more resourceful way.

Based on this knowledge, participants are asked to create their own ‘fun things to do’ list where they have to identify highly enjoyable activities which they can do on a regular basis in each week.^2^ This activity also helps to get them into a positive mind-set which subsequently helps them to reflect on their goals in such a way that they see more opportunities within their goals, feel more optimistic about pursuing their goals. Thus, it is assumed that identifying fun activities during the week will help participants to approach their goals with a more positive mind-set.

The second pleasure exercise builds on research around implicit-explicit motives alignment (Brunstein et al., 1998; Deci & Ryan, 2000). This strand of research states that there are two independent motivational systems which are considered independent from each other. The explicit motive system is very much based on people’s value system, conscious belief, self-image, etc. which then leads to the setting of explicit goals (Kehr, 2004). Our implicit motive system constitutes our relatively stable but unconscious needs that represent our affective preferences (McClelland, 1985). Research has conclusively shown that people differ in their capability to align or integrate those two systems with each other (Kehr, 2004; Sheldon, 2014) and the more people align their goal pursuit with their implicit motives, the more a goal is experienced as pleasurable. The three most widely researched motives in relation to implicit-explicit motive discrepancies are the achievement, affiliative and power motive. The HTGST draws on this strand of research and after introducing participants to the core characteristics of each of the three motives asks them in the first instance to self-assess^3^ their implicit motive strength in relation to the achievement, affiliative and power motive and – where appropriate – to realign their (explicit) goals towards their implicit motives. The exercise on implicit-explicit motive discrepancies, as well as the exercises on ‘fun activities during the week’, very much echoes the view of other Positive Psychology scholars who stress that pursuing happiness is about listening to the messages of our emotions and changing our behaviour (including goal pursuit), as it is about creating more pleasant emotions in the way we interact with the world (Bergsma, 2000).

### Altruism

The importance of altruism as an important goal-striving reason is discussed within the HTGST by drawing on research findings that demonstrate the positive impact of helping others for one’s own happiness (Dunn et al., 2008; Lyubomirsky, 2008). After participants have been introduced to the importance of helping others, the HTGST provides some practical guidance on how to integrate ‘helping others’ into one’s own life. In this context, research around *acts of kindness (AoK)* provides clear practical guidelines on how to do so. For example, Lyumbomirsky (2008) showed that performing a variety of different AoK contributes more to people’s happiness than performing the same AoK over and over again. Participants are also informed about the positive effect of remembering past AoK on happiness (Ko et al., 2019). After participants have been informed about how to perform AoK they are provided with a wide variety of possible AoK. Drawing on this list they are then asked to create their own list of about 15 AoK which they are encouraged to integrate into their daily lives.

Following on from the exercise on Aok, participants are then asked to reflect on the positive impact of their four most important goals for others. The HTGST draws hereby on the concept of job crafting which is defined as the ‘physical and cognitive changes individuals make in the task or relational boundaries of their work’ (Wrzesniewski & Dutton, 2001), p. 179). Particular focus is hereby given to cognitive job crafting where individuals reframe the meaning or purpose of their tasks in a way that makes a job more meaningful. Although cognitive job crafting is typically applied to a person’s job role as a whole, it is equally applicable to people’s most important goals. Essentially, cognitive job crafting encourages individuals to cognitively reframe (Ashforth & Kreiner, 1999) the significance of their goals by considering the impact of their goals on a higher level. Within the HSGST participants are therefore required to complete a worksheet whereby they need to state for each of their four most important goals the positive impact of this goal for others, the community, society or the environment. The overall aim of this exercise is therefore to increase participant’s self-awareness for the positive impact their goal pursuit can have on others.

### Necessity

The last part of the HTGST focusses on reducing people’s tendency to overestimate the necessity for material wealth as a means to increase happiness. Thus, participants are firstly introduced to the large body of literature that conclusively shows that once we reach a certain level of material wealth any additional increase in wealth is only marginally contributing to further increases in happiness (Diener & Biswas-Diener, 2002; Diener & Oishi, 2000; Kahneman et al., 2006). Participants are also informed about the substantial body of research findings which show that a strong focus on material wealth is typically negatively associated with happiness (Kasser & Ryan, 1993; Van Bowen, 2005). The information provided is used to stipulate critical reflections in relation to the question of what each participant deems necessary material wealth for themselves. To this end, participants were asked to complete an exercise in which they were required to reflect on the level of wealth they deemed sufficient to lead a happy life. During this exercise participants were also asked to reflect on the degree to which their four most important goals are driven by the (false) belief that happiness can be (strongly) increased through the accumulation of further material wealth. The exercise therefore allowed participants to reflect on their underlying motivation behind their most important goals, potentially redirecting their focus away from the accumulation of further material wealth and more towards pursuing their most important goals out of pleasure or for altruistic reasons. Given that many of these materialistic aspirations (i.e. bigger house, a more expensive car) are quite often associated with goal pursuits that are experienced as highly stressful, a reduction in those aspirations for further material wealth is quite often associated with reducing the amount of stress experienced during goal pursuit.

Finally, the training finished with an exercise on social-comparison (Festinger, 1954) as another reason why people want to accumulate more material wealth. This desire is not about satisfying the need for material wealth but to avoid having less than others. This element of the HTGST is based on research by Lyubomirsky and Ross (1997) which shows that happy people tend to perform less (negative) social comparisons than unhappy people (cf. Kim et al., 2016). Based on this research, participants were presented with the strategy of ‘internal standard setting’ rather than comparing oneself with others (Lyubomirsky & Ross, 1997) as a means to avoid social comparison where possible and focus on one’s own individual progress.

## Methods

### Procedure

The evaluation of the HTGST is based on two delivery modes (face to face, online). The face-to-face version of the training consisted of a one and a half day short course whereas the online training course lasted 6 weeks. In both cases, the effectiveness of the training was measured with a before-and-after study design whereby participants completed a pre-intervention questionnaire about 10 days prior to the start of the training and a post-intervention questionnaire about 2 weeks after the training. The face-to-face delivery of the workshops was conducted between March and August 2019. The online version was delivered between the 2nd of November and the 12th of December 2020 which was during the second wave of the Covid-19 pandemic in the UK where most participants of the training resided at the time.^4^ The online provision of HTGST was therefore one of many positive psychology practices that aimed to help individuals to cope with and grow through the imposed governmental changes such as stay-at-home orders including school closures and social distancing requirements (Walters et al., 2021).

Within both delivery modes participants were aware that their responses were part of a study, however, emphasis was placed on the practical use of the intervention for participants rather than on the research aspect of the study. Because of this, participants received an individual report at the beginning of the training informing them of their pre-intervention scores on the main study variables. Prior to the study, ethics approval from the relevant research institution was obtained for both delivery modes. To be eligible to take part in the study, participants had to be employed (full time or part time) or self-employed and between the age of 18 and 65. Participants were recruited by advertising the Happiness through Goal-Setting training through various internal and external university channels (open lectures to the public, social media, announcement in newsletters of the PI’s research institution) resulting in a heterogeneous pool of participants ranging from external employees from private and public sector organisations, members of staff from the PI’s own research institutions from various faculties as well as support staff but also as from selected part time students who study alongside their work commitments (Global MBA, MA HRM and Professional Doctorate in Coaching and Mentoring). This resulted in a wide variety of participants with heterogeneous backgrounds. Participation in the training was voluntary and not financially rewarded through any form of incentives.

### Participants

There were *N =* 41 people who completed the face-to-face version of the HSGST and *N =* 40 who completed the online version of the training. The gender distribution of participants attending the face-to-face delivery of the training was 80% female and 20% male with an average age of 43 (*SD =* 11.75). Similarly, gender distribution on the online version was 87% female to 13% male with an average age of 43 years (*SD =* 12.55). In the face-to-face version of the training, 88% held a permanent position whereas 12% were on temporary contracts. In the online version of the HSGST, 97% of participants reported being in a full time position and only 3% were on temporary contracts. In both delivery modes, there were slightly less people with management responsibilities than without management responsibilities (face-to-face version 29% to 71% and online version 42% to 58%, respectively).

### Measures

*Goal-striving reasons* were measured using the goal-striving reasons framework (Ehrlich, 2012; 2019; 2020) based on people’s four most important, self-selected, work-related (idiosyncratic) goals. Items are all preceded by the following text: ‘I strive for this goal because…’. Examples of items for each of the four goal-striving reasons are ‘I enjoy working on this goal a lot (pleasure)’; ‘It helps others’ (altruism); ‘If I fail I would feel like a loser’ (self-esteem); and ‘It is necessary to earn a living’ (necessity). Participants were required to answer all items on a seven-point Likert scale ranging from 1 = not true at all to 7 = very true. Shortly before the start of the online delivery of the HTGST, a validated short version of the goal-striving reasons questionnaire was developed (see Ehrlich, 2020). Because of this, the short form of the goal-striving reasons questionnaire was employed to evaluate the effectiveness of the online delivery of the HTGST. Both the long and the short form of the goal-striving reasons questionnaire are reported with high internal reliability (cf. Ehrlich, 2020) ranging from .88 to .94 for the long version and from .75 to .87 for the short version for all four goal-striving reasons. The internal reliability for the overall *goal-striving reasons index (GSRI),* a measure for the strength of people’s approach reason (pleasure, altruism) in relation to their avoidance reasons (self-esteem, necessity) were with .94 for the long version and .84 for the short version similarly high.

*Cognitive subjective well-being* was measured using the five item *Satisfaction with Life Scale (SWLS)* by Diener et al. (1985). The scale is reported to have strong internal reliability (Diener & Seligman, 2002). Participants need to answer all five questions on a seven-point Likert a scale ranging from 1 = strongly disagree to 7 = strongly agree.

*Affective subjective well-being* was measured using the short form of the PANAS scale by Watson et al. (1988) whereby participants have to answer to what degree they felt each of 10 positive affects (e.g. active, enthusiastic) and each of 10 negative affects (e.g. sad, depressed) within the last month. Participants are required to state how much they felt each of these affects employing an answer scale from 1= very slightly or not at all to 5 = extremely. The PANAS scale is reported in the literature with high internal reliability (Watson, et al., 1988).

*Work engagement* was measured using the short form of the Utrecht Work-Engagement Scale (UWES). Examples of items are ‘At my work I feel bursting with energy’ or ‘My job inspires me’. The answer scale ranges from 1 = never to 7 = always/every day. Internal reliability for this short form is also reported as high (Schaufeli & Bakker, 2003).^5^

## Results

The descriptive statistics in Table 1 show that participants in both samples (face to face, online) reported before the training slightly higher approach goal-striving reasons than avoidance goal-striving reasons, resulting in a positive GSRI in both cases. Differences in GSRI between both groups were not statistically significant (*M*_*f2f*_*=* 1.47; *SD =* 3.00; *M*_*online*_*=* 1.99; *SD =* 2.68; *t (79)* = −0.70, *p =* .48). Equally, both samples did not differ significantly on the main outcome variables of PA (*M*_*f2f*_*=* 3.45; *SD =* 1.00; *M*_*online*_*=* 3.50; *SD =* .78; *t (79)* = 0.20, *p =* .83), NA (*M*_*f2f*_*=* 2.37; *SD =* .91; *M*_*online*_*=* 2.35; *SD =* .82; *t (79)* = −0.35, *p =* .72) and work-engagement (*M*_*f2f*_*=* 4.93; *SD =* 1.27; *M*_*online*_*=* 4.84; *SD =* 1.06; *t (79)* = −0.30, *p =* .76). However, both samples did differ significantly with regards to life satisfaction where participants of the face-to-face training reported much lower levels of life satisfaction (*M*_*f2f*_*=* 2.98; *SD =* 1.01; *M*_*online*_*=* 4.58; *SD =* 1.39; *t(79)* = 5.84, *p <* .01) than the sample attending the online training. Performing paired sample t-tests revealed that in both delivery modes all outcome variables did significantly improve through HTGST. Admittedly, this effect has initially not been found with regards to altruism for the online version of HTGST due to nine individuals scoring very high on altruism prior to the training (*M* = 5.72; *SD* = .78) and much lower after the training (*M* = 3.87; *SD* = 1.31). Only after the elimination of these nine individuals did the training show some significant improvement on altruism. A rationale for the elimination of these specific individuals is provided in the discussion section.

**Table 1.** Differences in Main Study Variables Before and After the Training (Paired t-test).

	Face-to-face training						Online training					
	Before training		After training				Before training		After training			
	*M*	*SD*	*M*	*SD*	Δ	*Cohen’s d*	*M*	*SD*	*M*	*SD*	Δ	*Cohen’s d*
1) Goal-Striving Reasons Index	1.47	3.00	4.56	3.00	3.08**	1.16	1.99	2.68	3.85	2.41	1.85**	.74
2) Pleasure	4.56	1.13	5.32	1.01	0.76**	.82	4.67	.97	5.32	.91	0.64**	.63
3) Altruism	4.83	1.37	5.19	1.04	0.35*	.39	4.98	1.13	5.40	.92	0.42**	.53
4) Self-esteem	3.83	1.37	2.69	1.20	1.14**	.93	3.47	1.41	2.70	1.30	0.76**	.57
5) Necessity	4.09	1.52	3.27	1.33	.81**	.63	4.35	1.19	3.81	1.32	0.54**	.52
6) Life Satisfaction	2.98	1.01	4.55	1.37	1.56**	1.73	4.58	1.39	5.08	1.23	0.50**	.43
7) Positive Affect	3.45	1.00	3.64	.85	0.19*	.37	3.50	.79	3.80	.69	0.30*	.39
8) Negative Affect	2.37	.91	2.05	.72	0.32**	.50	2.35	.82	1.99	.66	0.36**	.45
9) Engagement	4.93	1.27	5.10	1.23	0.17*	.31	4.84	1.06	5.20	.97	0.35*	.39

*Note.* Cohen’s d is based on Morris’ (2008) procedure which takes into the account the correlation between pre- and post-test for each outcome variable. The evaluation of the online training revealed that nine people scored higher in altruism before the training then after. These nine individuals were eliminated for the analysis of altruism. Reasons for doing so, are elaborated on in the discussion section.

Overall the results of both evaluation studies show – as intended by HTGST – that both approaching reasons (pleasure, altruism) significantly increased through the training whereas the two avoidance reasons significantly decreased. Given that GSRI represents an accumulative score which reflects the overall change in all four goal-striving reasons the changes in GSRI before and after the training are the highest.

The effectiveness of the HTGST has also been analysed for people who prior to the training scored high or low on the main study variables. This is because related research has shown that people who initially score rather low on the trained variables typically tend to benefit proportionally more from PPI’s (Bergsma et al., 2020). Based on mean split, the sample was divided into participants having scored either above or below average on each of the nine main study variables before the training. These two groups were then compared using independent t-tests, on their relative gain on each of the main study variable before and after the training. To this end, a difference score was created whereby the scores of each participant before the training was subtracted from the score after the training. Thus, a positive difference score indicates an increase in any of the goal-striving reasons or outcome variables whereas a negative score indicates a reduction of any of the variables through the training (Table 2). The results clearly show that participants with lower scores prior to the training benefited significantly more from the training, in both delivery modes.

**Table 2.** Descriptive Statistics Showing Differences in Training Effectiveness Based on High/Low Scores on Main Variable Before the Training.

	Increase/decrease in scores on main study variables before and after the training									
	Face-to-face training				Online training					
	Low before the training		High before the training			Low before the training		High before the training		
	*M*	*SD*	*M*	*SD*	Δ	*M*	*SD*	*M*	*SD*	Δ
1) Goal-Striving Reasons Index	4.20	2.56	1.50	1.86	+2.70**	2.86	2.20	.68	2.02	+2.18***
2) Pleasure	1.27	.72	.38	.81	+0.82**	1.06	.86	.28	.96	+0.78*
3) Altruism	.84	.85	−.06	.61	+0.91**	.30	1.52	−.40	.90	+0.71*
4) Self-esteem	−1.55	1.24	−.71	.91	−0.85*	−1.26	1.12	−.23	1.26	−1.02*
5) Necessity	−.145	1.09	−.15	.99	−1.2**	−.85	1.01	−.15	1.04	−0.69
6) Life Satisfaction	1.61	1.53	1.52	.51	+0.08	.85	1.24	.12	.76	+0.76*
7) Positive Affect	.44	.58	.02	.33	+0.42*	.63	.63	−.03	.66	+0.66**
8) Negative Affect	−.58	.71	−.10	.36	+0.48*	−.84	.77	.02	.39	−0.87**
9) Work Engagement	.30	.70	.05	.35	+.025	.76	.95	−.09	.54	+0.86**

*Note.* Due to low sample sizes significance testing for delta (Δ) has been conducted using non-parametric methods of analysis (Mann–Whitney U Test).

## Summary and Discussion

Overall the results of both evaluation studies suggests that the HTGST is effective in improving people’s goal-striving reasons as well as having a positive impact on important outcome variables all representing positive psychological functioning (Life satisfaction, positive affect, negative affect and work-engagement). Generally, this was the case whether the training was delivered in a 1.5 days face-to-face workshop or online over a 6 weeks period whereby in both cases, the magnitude of changes was largely similar. Analysing the effect of the HTGST more specifically, it can be noted that the average effect sizes were slightly higher for the four goal-striving reasons compared to the effect sizes for the outcome variables which is not surprising giving that the HTGST contained exercises that were directly aimed at improving these specific goal-striving reasons. The reported effects sizes with regards to the four outcome variables are largely in line with the average reported effects sizes for PPIs ranging from *ds*.20 to .34 (see Bolier et al., 2013).^6^

The data also strongly suggests that participants with low goal-striving reasons or low levels of positive psychological function gained significantly more from the training than participants who scored above average on these variables. Again, these findings are in line with a variety of evaluation studies on positive psychology interventions (Bakker et al., 2020; Chan, 2010; Coote & MacLeod, 2012) all reporting that the gain from positive psychology interventions typically increases more so if the before-intervention scores on relevant outcome variables are low (Bergsma, et al., 2020).

With regards to altruism, the evaluation of the online training revealed that for a very small group of individuals, HTGST leads to a considerable reduction in altruistic goal-striving reasons. This fits with research indicating that specific subgroups benefit less from specific positive psychology interventions (Sheldon & Lyoburmisksy, 2019) which due to the negative publication bias towards non-significant findings (Bolier et al., 2013) still results in significant gaps in our understanding of how individual differences impact on the benefits of PPIs for certain individuals (Thompson et al., 2014). In this particular case, it is important to note that for this specific group of individuals altruistic goal-striving reasons did not stay the same before and after the training but were considerably reduced. This particular pattern of change in altruistic goal-striving reasons suggests that the pre-intervention scores were to an extent driven by selfless altruism (putting the needs of others before one’s own). This form of altruistic goal pursuit however contradicts the underlying rationale of the HTGST which promotes a form of altruistic goal-pursuit that promotes self-care alongside being altruistic. Thus, in this instance, it seems reasonable to assume that a reduction in altruistic goal striving can be seen as beneficial from a happiness perspective.

### Limitations

The findings of this study have to be treated with care because of the following limitations. Firstly, participation in both studies was voluntary and therefore both studies were open to a potential self-selection bias. Typically, voluntary participants of positive psychology interventions yield higher effects because participants are more motivated but also hold more positive opinions about happiness-increasing interventions (Bergsma et al., 2020). Thus, whether the training has the same positive effect for non-voluntary assigned participants remains to be seen.

Also, in both evaluation studies, the research design did not include a (randomised) control group design. The lack of a control group potentially allows for the possibility for a placebo effect to have taken place (Franke & Kaul, 1978) which means that the observed improvements before and after the intervention might have been partly due to the fact that participants were being part of an intervention rather than the content of the intervention. Whilst the possibility of a placebo effect cannot be ruled out due to the employed research design, the fact that the effects sizes of the HTGST are in line with findings from other PPIs restores some degree of confidence that the observed improvements are to a large extent due to the content of the interventions. Also, neither sample was balanced in terms of gender with a larger proportion of female participants. This potentially could also have impacted on the findings given that there is some evidence that indicates that there are gender specific effects that determine how much individuals benefit from different PPIs (cf. Crossley & Langdridge, 2005; Kashdan et al., 2009; Peura & Gayton, 2012). At the same time, research on the overall impact of happiness interventions suggests that women overall do not benefit more from PPIs than men (see Bergsma, et al., 2020).^7^ Given that the Happiness through Goal-Setting training is a multi-intervention approach, it is reasonable to assume that the overall effects of the training are not likely to be overly affected by the fact that both samples had a higher proportion of female participants. Finally, conducting multiple paired sample t-tests within the same sample increases the risks for type 1 error resulting in a higher likelihood that some of the significant difference in positive psychological functioning are due to chance rather than due to the effect of the intervention. However, in this particular study this risk seems relatively low as correcting for type 1 error, employing the Bonferroni adjustment procedure, would require a more conservative significance level of *p* < .01 which 13 out of the 18 paired sample t-tests results still meet. Furthermore, given that the findings are relatively similar between the two independent samples (f2f and online), the significant improvement in positive psychological functioning are very unlikely to be due to chance.

### Implications for practice

Despite these limitations the finding have important implications for practice. As research has conclusively shown, people’s SWB can be improved by modifying the reasons why people strive for their goals (Sheldon et al., 2004). Until now, those modifications were mostly focussed on helping individuals to pursue their goals for more autonomous reasons as suggested by self-concordance theory (Sheldon, 2014). The findings of this study suggest that it is equally relevant to help individuals to pursue their goals for more approaching (and less avoidance driven) reasons. With this in mind, the HTGST offers new and unique insights as well as exercises that help people to modify their goals towards more approaching reasons that are independent from the question whether people do so for autonomous or controlled reasons. For example, it is possible to increase the amount of pleasure experienced during goal-pursuit – even for goals that are pursued for more controlled reasons. Equally, one can easily imagine to reduce the fear of self-esteem loss associated with the pursuit of a goal even for goals that are pursuit for more autonomous reasons. These two examples demonstrate the unique contribution of the HTGST to help people change their reasons for goal pursuit – and ultimate improve their SWB – which goes beyond the currently available offer of autonomy-oriented, goal-reason focussed interventions.

### Future research

The results of this study also suggest future research. Firstly, it seems important to conduct further follow-up studies which employ a randomised control group design to eliminate any concerns that the reported positive impact of the HSGST may be partially due to a placebo-effect. Such future studies should also include a follow-up measure about 3 month after the intervention in addition to a post-intervention measure.^8^ Furthermore the two presented evaluation studies focussed on the overall effect of the HTGST and therefore did not analyse more specifically, largely due to the given sample size, which of the exercises worked best for which particular individuals. According to Sheldon and Lyubomirsky (2019) it is an important contribution to the positive psychology literature to show the differential effects of various positive psychology interventions for various subgroups and under various conditions which also includes the identification of relevant moderation and mediation effects. An ideal starting point seems to be to look into specific characteristics of those individuals that seem not to benefit from the altruism exercises of the training due to extremely high pre-intervention scores on altruism. In particular, the hypothesis that those individuals might be the ones with an overly high degree of self-sacrificing altruistic goal pursuit seems important to analyse further.

With regards to effectiveness of the HTGST it also has to be noted that in both evaluation studies participants were rather mature with an average age of 43 years. Research suggests that mature individuals benefit more from PPIs as they are more self-reflective (Bergsma et al., 2020; Sheldon & Lyubomirsky 2019). Thus, future studies are needed to establish whether the HTGST can replicate the positive findings when using younger participants, for example, UG students or even secondary school children.

## Conclusion

To conclude, the HTGST is an effective multiple intervention training approach grounded in a unique goal-oriented theoretical framework: the GSRF. Because of the comprehensive nature of the GSRF comprising of four important goal-striving reasons (Pleasure, altruism, self-esteem and necessity) it permits an integration of a multitude of training interventions which, according to a variety of scholars (Bergsma et al., 2020; Sheldon & Lyubomirsky, 2012) is preferable to single interventions as they prevent the occurrence of hedonic adaptation.

Furthermore, given its specific goal-focus the HTGST is particularly suitable for individuals with a strong goal-orientation, that is, individuals who prefer to organise their lives around the attainment of their most important goals. Here, the HTGST offers an opportunity to reflect and modify their reasons for goal pursuit. As the quality of reasons behind people’s goal pursuits is an important predictor of positive psychological functioning (K. M. Sheldon et al., 2004) the HTGST can be regarded as an important additional positive psychology intervention. To our knowledge, such a particular approach focussing predominantly on people’s reasons for goal pursuits does not yet exist.

## Note

1 The topic of unconditional self-esteem is hereby introduced to the participants before the other three goal-striving reasons as any reduction in fear of self-esteem loss is a good catalyst for the following goal-striving reasons as it reduces a strong avoidance motivation component within their goal-striving reasons allowing, so we argue, participants to develop their approach goal-striving reasons more fully (Pleasure, altruism).

2 At the same time, participants are given information about hedonic adaptation and the importance of variety within those fun exercises to avoid hedonic adaptation as much as possible.

3 Admittedly, this self-assessment is not based on a validated test such as the Multi-Motive Grid (Sokolowski et al., 2000) but serves to form a working hypothesis about one’s implicit motive strength. For the purpose of this exercise, this is deemed sufficient as the focus is on the modification of goals to make them more enjoyable. Thus, the ultimate criteria whether this exercise was applied correctly is in the anticipated increased pleasure after the goal would be executed in a way that is anticipated to be more enjoyable – the knowledge about the three implicit motive provides the theoretical framework for participants to think about which specific modifications to their goals are most likely to increase the amount of pleasure during goal pursuit.

4 Within the online training participants were randomly assigned to be either tutor-led (n = 24) whereby participants could ask question through an online forum or guided by a Digital Well‐Being Coach consisting of a specifically developed desktop chatbot application for the HTGST (n = 16). Non-parametric analysis (Mann–Whitney U test) revealed no significant differences between the two groups for the main study variables post intervention. Because of this, in addition to the small numbers in each group, no further analysis between tutor-guided versus app guided have been conducted.

5 Given that the online version of the HTGST was conducted during the Covid‐19 pandemic, it was anticipated that some part time students who could not pursue their normal work anymore might focus predominantly on their studies. For this particular group of participants, a student version of the questionnaire was offered which was mostly similar to regular employee survey but was based on people’s four most important goals at university as well as employing the student version of the Utrecht Work Engagement scale. An Independent-Samples Mann–Whitney U Test, revealed no significant differences between this particular group of participants and the participants who completed the employee version of the survey with relation to engagement or indeed all other main study variables.

6 With the exception of Life Satisfaction in the face-to-face mode where the pretest scores were very low, hence this sample reported much higher gains on life satisfaction through the training as explained in the next paragraph.

7 Bergsma et al. (2020)identified age but not gender as a factor that impacts on how much individuals benefit from PPIs.

8 In this context, it is also important to note that in the online delivery, the time lapse between pre-test and post-test was in effect 10 weeks compared to the face-to-face delivery where the time period between pre- and post-test was 4 weeks. In both cases, the effect sizes were reasonably similar providing some tentative support for the fact that a longer follow-up measure, for example, of 3 month, most likely reveals similar findings as reported in the study at hand.
